# The Report of the Recent Committee

**Published:** 1914-10-24

**Authors:** 


### A STANDARD METHOD FOR EXAMINING WATER.

### The Report of the Recent Committee.

The importance of the adoption of a standard method of procedure for the bacteriological examination of water has been carefully recognised both here and in America. In 1904 a committee of the Royal Institute of Public Health published recommendations dealing with the matter which have been generally adopted in England. In the United States of America a committee of the Public Health Association issued suggestions of a similar character in 1897, 1905, and in 1912. Having regard to advances which have been made in this department of work in recent years, the Royal Institute of Public Health in March 1914 appointed a new committee, under the chairmanship of Dr. A. C. Houston, the director of water examination for the Metropolitan Water Board, to reconsider the whole subject. This committee has recently presented its report.

This report, as might be anticipated, differs in several important respects from the report of 1904. Its recommendations are in accord with the opinions of most bacteriologists who have devoted special attention to the subject, and are likely to meet with general adoption. It is suggested that the following tests should be applied in the case of all water samples:—

1. The estimation of the number of microbes per cubic centimetre (*a*) in meat-broth gelatine incubated at 20° to 22° C.; (*b*) in meat-broth agar incubated at 37° C.
2. Tests for the presence of the *Bacillus coli*.

In special cases it is advised that the following additional tests should be employed:—

(1) The estimation of the number of microbes per cubic centimetre in lactose bile-salt agar incubated at 37° C. (2) Tests for streptococci. (3) Tests for the presence of *B. enteritidis* sporogenes. (4) Tests for pathogenic microbes.

The retention of the 20° count is of special interest in view of the fact that the American committee of 1912 recommended its discontinuance. The English committee's decision will meet with general approval. Even in America the proposal to discontinue this count has met with general opposition, and the laboratory section of the American Public Health Association, which appointed the committee responsible for recommending its disuse, passed a resolution in September 1912 that the count should be retained.

The amounts of water which should be plated for the counts, the time at which the counts should be made, and the precautions which should be taken in collecting samples are dealt with in full detail. The recommendations differ in some important respects from the similar recommendations of the 1904 report, the most important difference being the suggestion that the final count should be made at the end of 4 days instead of 3.

As regards the identification of the colon bacillus, the committee recommend the use of MacConkey's bile-salt peptone medium, with the addition of either glucose or lactose. They point out that some bacteriologists incubate their cultures at 37° C. and others at 42° C. On the whole, the committee prefer the former temperature. Suspicious colonies should be picked off the plates and tested for gas formation in a lactose-peptone medium and for indol in a peptone medium. The committee suggest that all reports should state the presence or absence of lactose +, indol + microbes, the smallest amount of water in which they were present, and the largest amount from which they were absent. They advise that samples of water containing glucose fermenting coli-like bacilli which give negative results with the lactose and indol tests should be viewed with suspicion. With regard to streptococci, the committee favour the Drigalski and Conradi medium for direct culture and the exclusion of all streptococci which do not yield acid in a lactose medium. They are of opinion that a water should not be condemned on the streptococcus test alone if the *Bacillus coli* and other results are satisfactory. In this respect they are in agreement with the American committee of 1912. The committee say they do not think it practicable to lay down a fixed standard of purity applicable to all cases. All they feel justified in stating is that the further a water departs from the standard of no lactose +, indol + *B. coli* in 100 c.c., the greater is the suspicion attaching to it, unless the local conditions exclude undesirable pollution.

### The Wording of Reports.

This confirms the view generally held by water analysts, that a bacteriological examination in itself —apart from the actual discovery of pathogenic organisms—is insufficient to determine the potability of a water. That the committee hold this view is further evidenced by the warning they give as to the wording of reports. They say: "It is undesirable, when the source and surroundings of the supply are unknown to the bacteriologist, that he should use statements such as: 'This water is grossly polluted, and is most dangerous for drinking purposes'; or 'This water is of superlative quality, and is absolutely safe for consumption.' He should express himself in more guarded terms, such as: 'This particular sample of water, as judged by the above tests, yields unsatisfactory (or satisfactory) results.'" All practical hygienists will agree with these cautious words. An opinion as to the potability of a water can be formed only after a careful consideration of the results of bacteriological and chemical examination, and of the local conditions of the supply. The great value of the bacteriological examination is that it may direct attention to the possible presence of pollution, which might otherwise be overlooked, and that in the case of a periodic examination of a given water supply it affords an additional test of its purity by a comparison with previous results.

